# The Uptake of Ethinyl-Estradiol and Cortisol From Water by Mussels (*Mytilus* spp.)

**DOI:** 10.3389/fendo.2021.794623

**Published:** 2021-12-15

**Authors:** Ioanna Katsiadaki, Tamar I. Schwarz, Alex R. O. Cousins, Alexander P. Scott

**Affiliations:** ^1^ Centre for Environment, Fisheries and Aquaculture Science, Weymouth Laboratory, Weymouth, United Kingdom; ^2^ Centre for Environment, Fisheries and Aquaculture Science, Lowestoft Laboratory, Lowestoft, United Kingdom

**Keywords:** depuration, endocrine disruption, mollusk, steroid, ethinyl-estradiol, cortisol

## Abstract

Previous toxicokinetic studies have shown that mussels (*Mytilus* spp.) can readily absorb the three main mammalian sex steroids, estradiol (E_2_), testosterone (T) and progesterone (P) from water. They also have a strong ability to store E_2_ and the 5α-reduced metabolites of T and P in the form of fatty acid esters. These esters were shown to have half-lives that were measured in weeks (i.e. they were not subject to fast depuration). The present study looked at the toxicokinetic profile of two other common steroids that are found in water, the potent synthetic oestrogen, (ethinyl-estradiol) (EE_2;_ one of the two components of ‘the pill’), and cortisol, a natural stress steroid in vertebrates. In the first three hours of uptake, tritiated EE_2_ was found to be taken up at a similar rate to tritiated E_2_. However, the levels in the water plateaued sooner than E_2_. The ability of the animals to both esterify and sulphate EE_2_ was found to be much lower than E_2_, but nevertheless did still take place. After 24 h of exposure, the majority of radiolabelled EE_2_ in the animals was present in the form of free steroid, contrary to E_2,_ which was esterified. This metabolism was reflected in a much lower half-life (of only 15 h for EE_2_ in the mussels as opposed to 8 days for E_2_ and >10 days for T and P). Intriguingly, hardly any cortisol (in fact none at all in one of the experiments) was absorbed by the mussels. The implications of this finding in both toxicokinetic profiling and evolutionary significance (why cortisol might have evolved as a stress steroid in bony fishes) are discussed.

## Introduction

**Figure 1:**
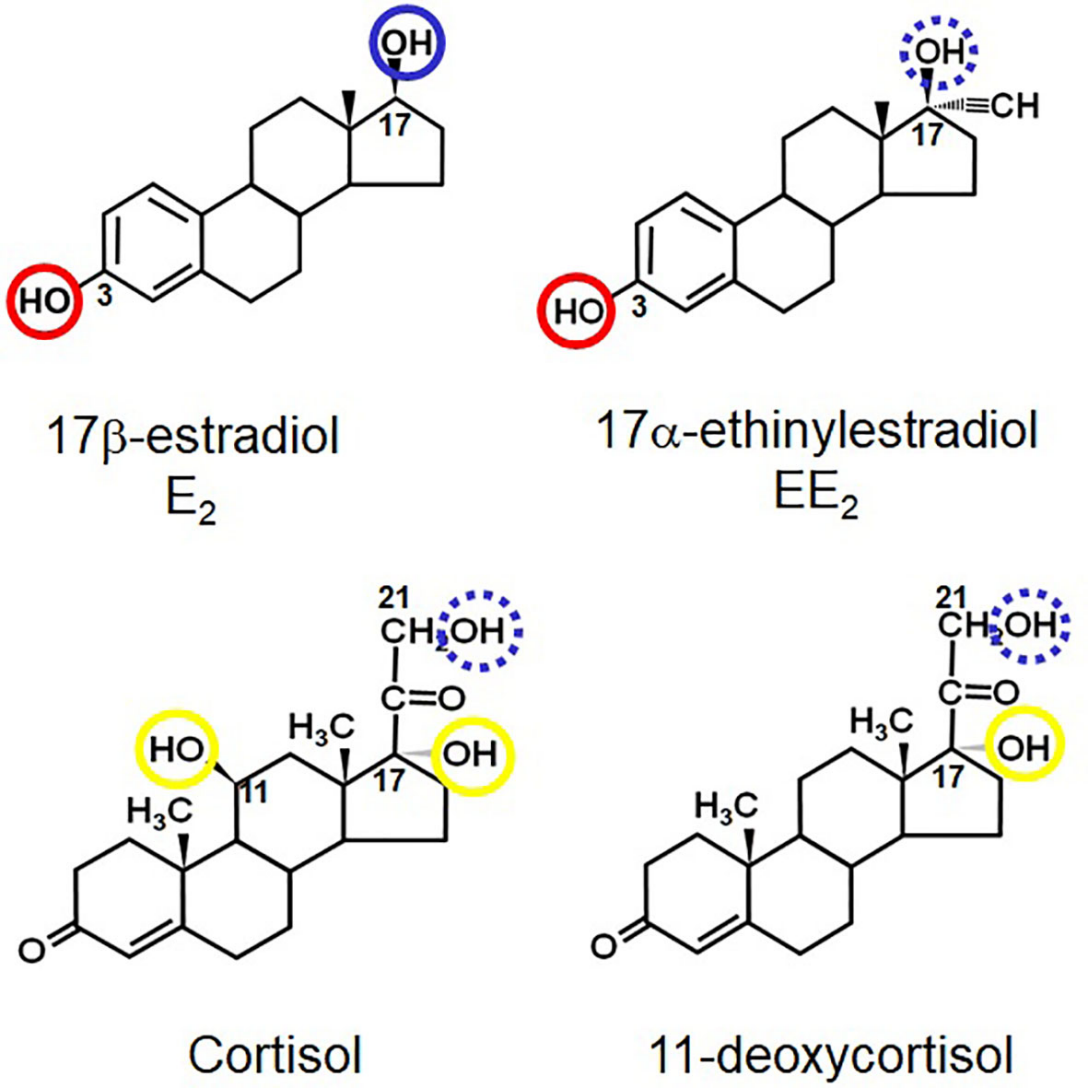
Structures of E_2_, EE_2_, cortisol and 11-deoxycortisol- highlighting the positions of key hydroxyl groups in all four steroids. Those circled in blue are known or hypothetical (e.g. the 21-hydroxyl group of cortisol) points of esterification. Those circled in red are where preferential sulphation occurs. Those circled in yellow cannot be naturally conjugated (i.e. they are non-reactive). The numbers show the relevant positions of the groups on the steroid skeleton.

There has been a multitude of studies on the potential role of vertebrate-type sex steroids in the reproduction of mollusks over the last seventy years (as reviewed by 1–4). Most of these studies, especially the earlier ones, have attempted to prove that the common sex steroids, 17β-estradiol (E_2_), testosterone (T) and progesterone (P) act as hormones in mollusks in the same way that they do in vertebrates. Measurement of sex steroids in mollusk tissues was commonly presented as evidence for the relevance of their hormonal role in the hope that changes in concentrations would correspond in an expected way with different stages of their reproductive cycle (e.g. E_2_ concentrations would be at their highest at the height of gonad maturation of females), sex (e.g. E_2_ concentrations would be higher in females than in males) and after treatment with endocrine disrupters (e.g. there would be an increase in T in response to Tributyl Tin exposure). Apart from the fact that any such evidence is circumstantial (correlation not being proof of cause and effect), several recent reviews have pointed out why such expectations are problematic (2–6). The review by Scott (4) in particular pointed out that, regardless of whether or not mollusks are able to make their own vertebrate steroids, there are three very important factors that preclude an unambiguous link between concentrations and reproductive processes in mollusks: firstly, the environment (that includes laboratories) is awash with vertebrate steroids (7); secondly, water-dwelling mollusks, especially bivalves, have a remarkable ability to absorb the common human sex steroids, P, E_2_ and T from water (8–10); and, thirdly, mollusks have an even more notable ability to store these steroids and/or their metabolites in the form of fatty acid esters (8–14). Esterification involves the conjugation (typically *via* removal of a water molecule) of the carboxyl group of a fatty acid with a reactive hydroxyl group of a steroid. Hydroxyl groups that have been shown to be targets for esterification are those found at the 17β position in T and E_2_ (**Figure 1**) and at the 3β position of certain 5α- and 20β-reduced metabolites of T and/or P. There is no evidence that the hydroxyl group at position 3 of E_2_ is conjugable with fatty acids, although it can be conjugated with a sulphate group (**Figure 1**). The uptake of steroids by mollusks and the fact that the steroids can be readily metabolised and conjugated is usually not taken into account by researchers who measure steroids in invertebrates - even though the notable ability of mollusks to absorb and esterify steroids was convincingly proved twenty years ago (11). Publications that give no consideration to the possibility that steroids in mollusks might be contaminants rather than endogenously produced hormones or a mixture of both continue to appear in the literature.

As mentioned above, our previous work has already described the pattern of uptake, metabolism and esterification of E_2_, T and P by the mussel, *Mytilus* spp. (8–10). We found that all three were taken up very readily by mussels, but metabolised in different ways. Most of the E_2_ was either esterified unmodified (and accumulated in the animal) or converted to a sulphate (which accumulated in the water). Testosterone was strongly esterified. However, analysis of both free and ester fractions showed that >90% had been converted to 5α-reduced metabolites. About a fifth of the tritium was also lost from the T radiolabel in the form of tritiated water, but there was no evidence that this was linked to E_2_ production. Progesterone radiolabel was also strongly accumulated in the ester fraction. However, this was entirely due to formation of 3β- and/or 20β-hydroxylated 5α-reduced metabolites. The purpose of the present paper was to investigate the uptake of two other steroids, ethinyl-estradiol (EE_2_; the estrogenic component of “The Pill”) and cortisol, which is abundant in natural waters, as it is the stress steroid of bony fishes. We hypothesised that EE_2_ would behave in a similar way to E_2_, but likely slower, due to the fact that the reactive β-hydroxyl group on the C17 position (which is where esterification takes place) is paired with an α-hydrogen atom in E_2_, but with an α-ethinyl group in EE_2_ (**Figure 1**). We hypothesised that this would diminish esterification, but not sulphation, as this was shown by us to occur in mussels on the hydroxyl group at the C3 position of E_2_. In the case of cortisol, because it has an unhindered primary hydroxyl group at the C21 position (**Figure 1**), we hypothesised that this would be a readily reactive site for esterification. In other words, we expected cortisol to build up strongly in the ester fraction.

## Materials and Methods

The experiments described here were part of a large series of experiments, all aiming at describing the toxicokinetic profile of common vertebrate steroids in the common mussel.

### Animals

Mussels were collected from two locations; The Retreat, Brancaster Staithe, Norfolk during March 2014 (Experiment 1, 50 animals) and Portland Harbour, Dorset during October 2014 (Experiment 2, 110 animals) and November 2015 (Experiment 3, 16 animals). Both areas are populated predominantly by *Mytilus edulis*, but we cannot discount the co-existence of *Mytilus galloprovincialis* and/or their hybrids (hence we refer in this paper to ‘*Mytilus* spp.’). The animals were acclimated to the experimental conditions for 6, 5 and 1 days for Experiment 1, 2 and 3 respectively. Animals were not sexed or aged. The average wet weight of the animals and the mean shell length mean varied between 3.16-4.62 g and 49-58 mm respectively in all experiments.

### Methods

The methodology and toxicokinetic profile has already been described for E_2_, T and P (8–10). All procedures involved placing the animals in containers with filtered (50 µm) seawater (with aeration or pre-aerated water), adding radiolabel to the water and then taking 1 mL water samples at various intervals in order to measure the amount of radioactivity remaining in the water. Sorption controls (no animals) were in place to measure any losses due to sorption or aerosol formation. Whole mussel soft tissue (i.e. not including the shell) was used for the analyses. Certain conditions employed in these experiments (i.e. duration, vessel type, volume of water) varied as part of method development. **Table 1** displays the experimental conditions relevant to the data reported here.

**Table 1:** Summary of conditions employed in each experiment.

Exp	Treatments	Vessel type	Animals per vessel	Vessel replicates	Water volume	Length of exposure
1	EE_2_ radiolabel only	Glass tank with rods	5	10	13L	2x 48h
	EE_2_ sorption & aeration control		0	1		
2	EE_2_ radiolabel only	Buckets lined with polythene bag	5	6	2L	24h
	EE_2_ low		5	2		
	EE_2_ high		5	2		
	EE_2_ sorption & aeration control		0	2		
	C radiolabel only		5	6		
	C low		5	2		
	C high		5	2		
	C sorption & aeration control		0	2		
	E_2_ positive control		5	2		
3	C radiolabel only	Polypropylene beakers	1	8	0.2L	6h
	C sorption control		0	8		
	11-Deoxy-C radiolabel only		1	8		
	11-Deoxy-C sorption control		0	8		

**Figure 2:**
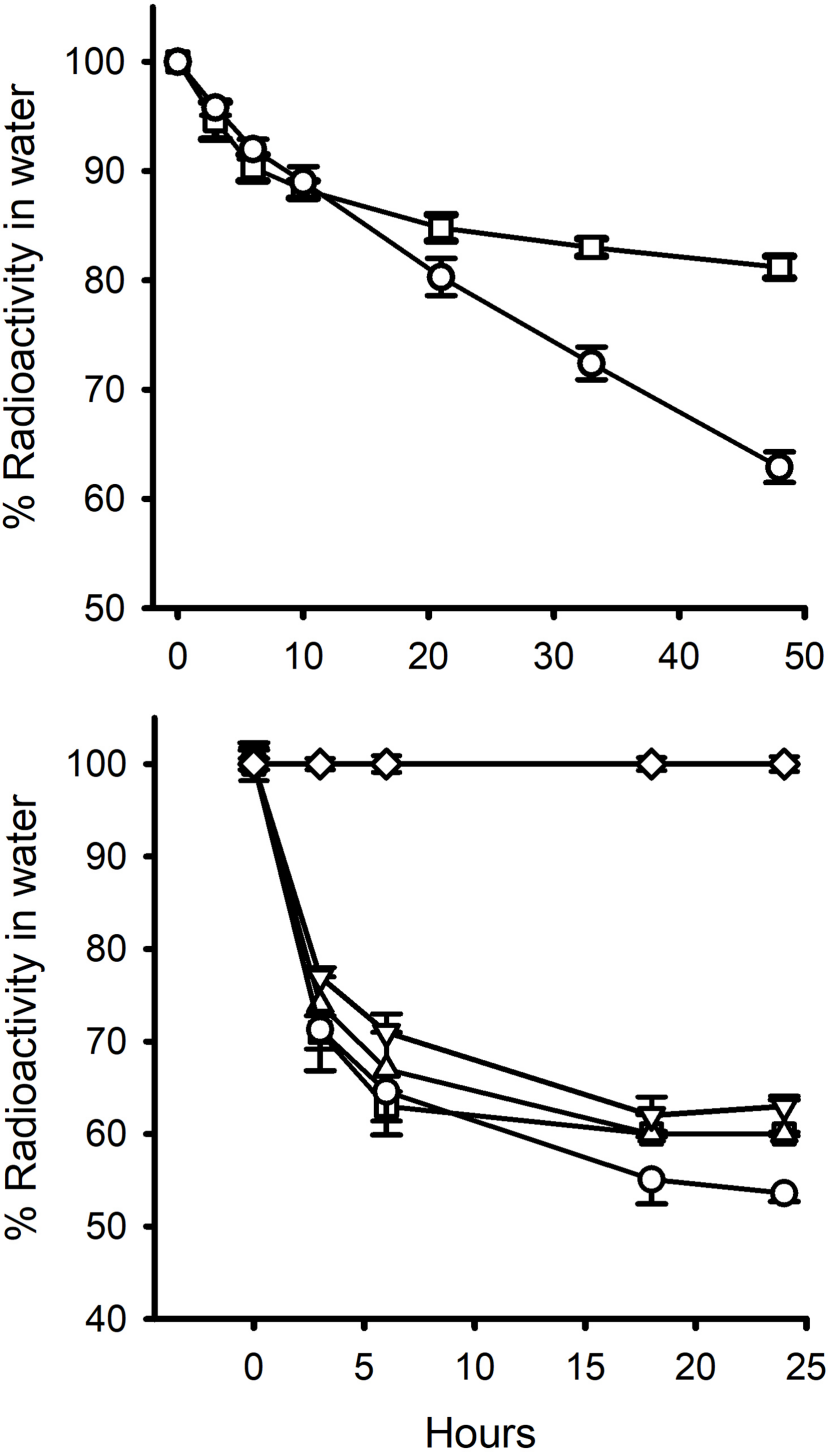
Percent of radiolabelled steroid removed from: top graph, 13L water containing 5 mussels over 48 h ( ± S.E.M; n=10) (Exp.1); and, bottom graph, 2L water containing 5 mussels over 24h (Exp 2.). ○, E_2_; □ EE_2_; ∆, EE_2_ +2.5 μg cold steroid; ▿, EE_2_ + 25 μg cold steroid; ⋄, Cortisol. Standard errors are also shown. In the 2L experiment, the proportions were corrected for non-specific loss of radiolabel in control tanks (ca. 9% by 24h for E_2_ and EE_2_ and 5% for cortisol in the 2L water experiments). Non-specific radiolabel loss in the 13L tank experiments was negligible (< 2%).

**Experiment 1**: The first experiment was carried out under identical conditions to *Exp. 1* in the publications on E_2_ (8, 15), which are displayed in **Table 1**. The animals were transported in a cool-box overnight and immediately placed in a flow-through system of sea water. Individual rods were placed vertically in aerated cylindrical glass tanks with 13 L of sea water at 16 ± 1°C with a 16:8 h light:dark photoperiod for exposure to radiolabel. Five mussels were added in each tank. The animals were fed Shellfish Diet^®^ 1800 (a mix of *Isochrysis* spp., *Pavlova* spp., *Tetraselmis* spp., *Thalassiosira weissfloggi* and *Thalassiosira pseudonana*) following manufacturer’s instructions and the water was changed daily. The mussels were dosed with 0.86 μCi L^-1^ (4.25 ng L^-1^) ethinyl-estradiol, 17-[6,7-^3^H(N)] ([^3^H]-EE_2_) purchased from American Radiolabeled Chemicals, Inc. (101 ARC Dr. St. Louis, MO 63146 USA). Samples were collected at intervals (see **Figure 2** for frequency) over 48 h. The animals were given a second dose of radiolabel and kept for another 48 h to build up the amount of radiolabel in the tissues and provide sufficient material for subsequent metabolite analysis. For depuration, animals were placed in fresh water in shallow trays under flowthrough conditions (1 L min^-1^). Ten animals were sampled on day 0, 5, 10, 15 and 20.

**Experiment 2**: The second uptake experiment was run under identical conditions to *Exp. 4* in the publication by Schwarz et al. (8) and details are provided in **Table 1**. Briefly, five mussels were placed in an aerated bucket lined with a polyethylene bag and filled with 2 L of filtered sea water at 16 ± 1°C with a 16:8 h light:dark photoperiod for exposure. Water was changed daily, and animals were fed Shellfish Diet^®^ 1800 daily (following manufacturer´s instructions). The animals were given the same amount of [^3^H]-EE_2_ (2.9 µCi L^-1^), but had either no (n = 6), 2.5 µg L^-1^ (low; n=2) or 25 µg L^-1^ (high; n=2) of cold EE_2_ added. There were also bags in which mussels were exposed to [^3^H]-E_2_ (2.74 µCi L^-1^, n=2, used as a positive control), to [^3^H]-cortisol (3.06 µCi L^-1^) with no (n=6), 2.5 µg L^-1^ (low; n=2) or 25 µg L^-1^ (high; n=2) of cold C and to [^3^H]-EE_2_ (3.45 µCi L^-1^, n=2) and [^3^H]-cortisol (3.29 µCi L^-1^, n=2) only (sorption controls; i.e. no animals). Water (1 mL) samples were taken at 0, 3, 6, 18, 24 h from all vessels and immediately placed in scintillation fluid for counting (see **Figure 2**, bottom graph). For depuration, animals were placed in fresh sea water in shallow trays under flowthrough conditions (1 L min^-1^). Ten animals were sampled on day 0 and five animals were sampled on day 5, 10, 15 and 20.

**Experiment 3**: The animals were transported in a cool-box and kept for at least 24 h in running seawater before exposure experiments. The temperature was not controlled during exposures and ranged between 15-19°C. The animals were not fed as exposures were short. Individual mussels were placed in polypropylene beakers with 200 mL pre-aerated filtered seawater for six hours with either [^3^H]-cortisol (3.8 µCi L^-1^) or [^3^H]-11-deoxycortisol (6.08 µCi L^-1^) and an equal number of sorption controls for each steroid. No aeration was carried out during the 6 h exposure period. Water samples (1 mL) were collected at regular intervals and immediately mixed with 7 mL scintillation fluid. After exposure, all animals were frozen at -20°C for later extraction. No depuration followed Experiment 3.

### Metabolite Separation and HPLC

The methods for extracting and separating free and esterified steroids (and HPLC conditions) have all been previously described in the above-mentioned papers.

### Clearance Rates

The rates at which individual mussels initially cleared steroids from water (i.e. clearance rates) were calculated as follows. The percentage radiolabel remaining in the water from each treatment were first of all corrected (if necessary) for loss of radiolabel due to sorption. Label disappearance data were fitted to hyperbolic decay curves using Sigmaplot (Systat Software Inc, TW4 6JQ, London, UK.) as described previously (8). In all cases, except for cortisol in Experiment 3, *r* ^2^ was >0.99. The calculated proportion of radiolabel that had been removed from the water by 1.5 h was used to derive a rough estimate of the ‘clearance rate’ of an individual mussel (mL animal^-1^ h^-1^) at the start of each exposure period:


$$Initial\;clearance\;rate=\frac{rV}{1.5n}$$

Where *r* = proportion of radiolabel removed over the first *1.5* h; *V*= total volume of water in the container (mL); *n* = number of animals in the container.

## Results

### Uptake

In Experiment 1 (**Figure 2**) the reduction in the level of EE_2_ in the water was very similar to that of E_2_ over the first 3 h (8, 15) but then diverged. Ethinyl-estradiol plateaued at a higher level than E_2_. This same pattern was found in the second 48 h incubation period (**Table 2**). In Experiment 2, there was a marked decrease in radioactivity in all the bags containing mussels with either E_2_ or EE_2_, but zero decrease in the levels of [^3^H]-cortisol (**Figure 2**). There was a slight decrease in [^3^H]-EE_2_ and [^3^H]-cortisol (up to 9% and 5%, respectively, over 24 h) in the control bags with no animals. The amounts of radioactivity remaining in the water levelled off after 18 h at *c*. 50% for E_2_ and *c*. 60% for the three EE_2_ treatments. In the first 1.5 h, the calculated clearance rate (mL animal^-1^ h^-1^) of EE_2_ was higher (61.3) than that of E_2_ (52.0) (**Table 2**). The addition of large amounts of cold steroid appeared to reduce the clearance rates of EE_2_ to 49.6 and 41.6, respectively, but this was not statistically significantly different (due to the fact that there were only two containers each for the cold EE_2_ treatments). By the end of the experiment, the proportion of radiolabel that had been absorbed was the same for all three treatments (**Table 2**).

**Table 2:** Clearance rates at beginning of incubation period and percentage of radiolabel absorbed by end of incubation period.

		Clearance rate(mL animal^-1^ h^-1^)	% Radioactivity absorbed by end of experiment
Experiment 1 48 h	EE_2_ first 48 h	46.0	20
	EE_2_ second 48 h	43.3	13
	E_2_ first 48 h	44.8*	37
	E_2_ second 48 h	46.7*	28
Experiment 2 24 h	EE_2_ label only	61.3	40
	EE_2_ + low dose of cold	49.6	40
	EE_2_ + high dose of cold	41.6	38
	E_2_ label only	52.0	46
	Cortisol	0	0
Experiment 3 6 h	Cortisol	6.4	9
	11-deoxycortisol	17.4	26

*Data shown for comparison purposes only, as previously reported by Schwarz et al. (8).

**Figure 3:**
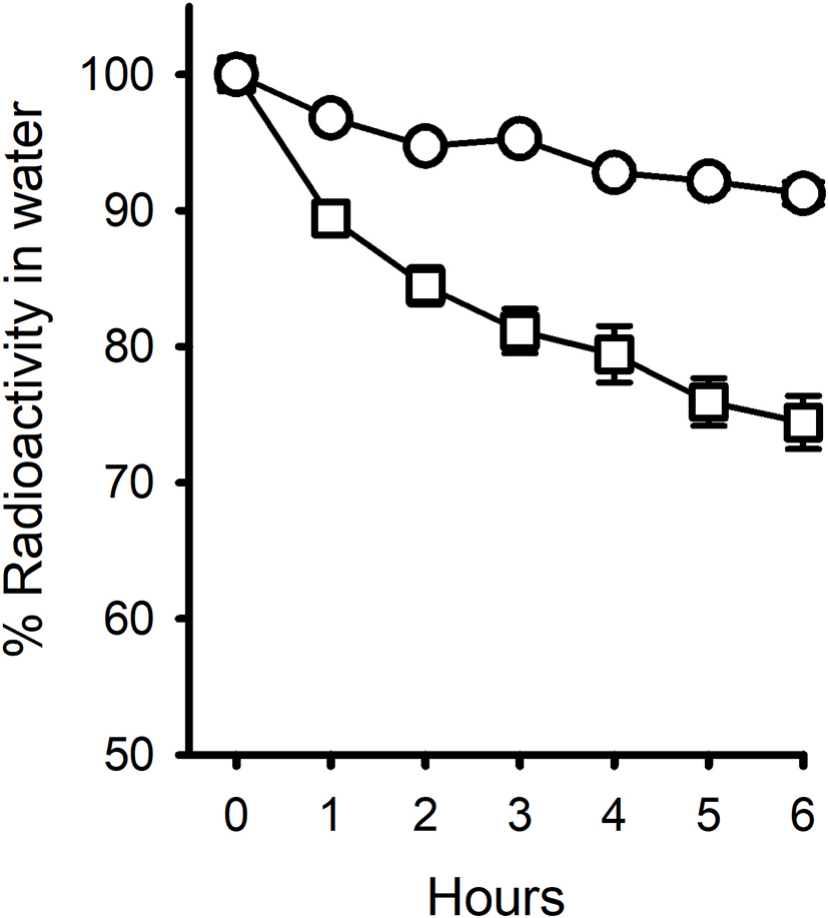
Pattern of disappearance of radiolabelled Cortisol (○) and 11-deoxycortisol (□) steroids from 200 mL water containing one mussel over 6 h. Each treatment had 8 replicates. Non-specific radiolabel loss was negligible (0.34% for Cortisol and 0.16% for 11-deoxycortisol). Error bars are shown.

In Experiment 3, cortisol decreased by about 5% over 6 h and 11-deoxycortisol by 25% (**Figure 3**). Because there was no aeration, there was no non-specific loss of radiolabel in control pots with no animals.

The efficiency of ethyl acetate in extracting EE_2_ radioactivity from tissue was established to be between 95-98%, the same as for E_2_ and T (8, 9). When ten of each of the mussels from the EE_2_ treatments (no depuration) were extracted (Exp. 2), a statistically significant difference was found in the concentration of radiolabel (pg g^-1^ wet weight; n=10; ± sem) between the treatments (radiolabel-only, 370 ± 30; low, 470 ± 10; high, 480 ± 10). The average weights of wet tissue animal^-1^ in the three groups were 4.09, 4.75 and 4.34 g. Basically, low and high amounts of cold EE_2_ appeared to increase rather than decrease the concentration of radiolabel that was extracted from the tissues at the end of the 24 h exposure period. This could be due to the saturation of detoxification capacity. Whatever the reason for the disparity in the water v. tissue results, the important conclusion is that microgram quantities of cold EE_2_ do not appear to saturate the uptake of radiolabelled EE_2_.

**Figure 4:**
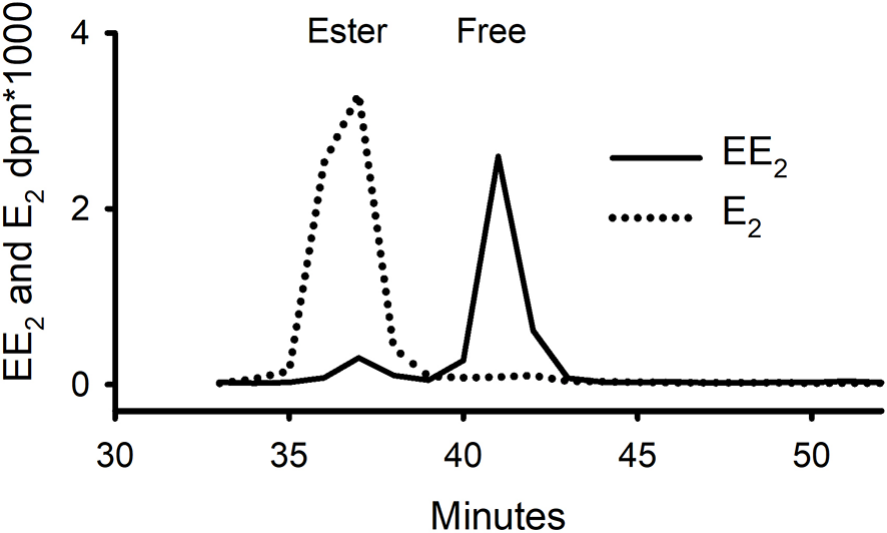
Separation on normal-phase HPLC of whole tissue extracts of mussels that had been exposed for 96 h to either tritiated E_2_ or EE_2_ (Exp. 1) showing contrasting distribution of radioactivity between free and ester fractions.

The solvent separation procedure (which involved partitioning the radioactivity twice between heptane and 80% ethanol) was also shown to work as well for EE_2_ as for E_2_, P and T (data not shown). The ratio of ester:free:water-soluble in the EE_2_ extracts (4:88:8) was strikingly different from that found for E_2_, in which the same ratio was 85:11:4, strongly indicating that the 17α-ethinyl group on EE_2_ obstructs the esterification of the 17β-hydroxyl group. To confirm that this was not an anomaly of the solvent separation procedure, some of tissue extract from mussels in Experiment 1, that had been dosed twice with [^3^H]-EE_2_ over a four-day period, was run on normal phase HPLC (**Figure 4**). This confirmed that there was a large peak of activity corresponding to free EE_2_ and only a small peak in the expected elution position of esterified EE_2_. The opposite situation was found for E_2_, where the majority of the steroid was in the form of ester.

### Depuration

**Figure 5:**
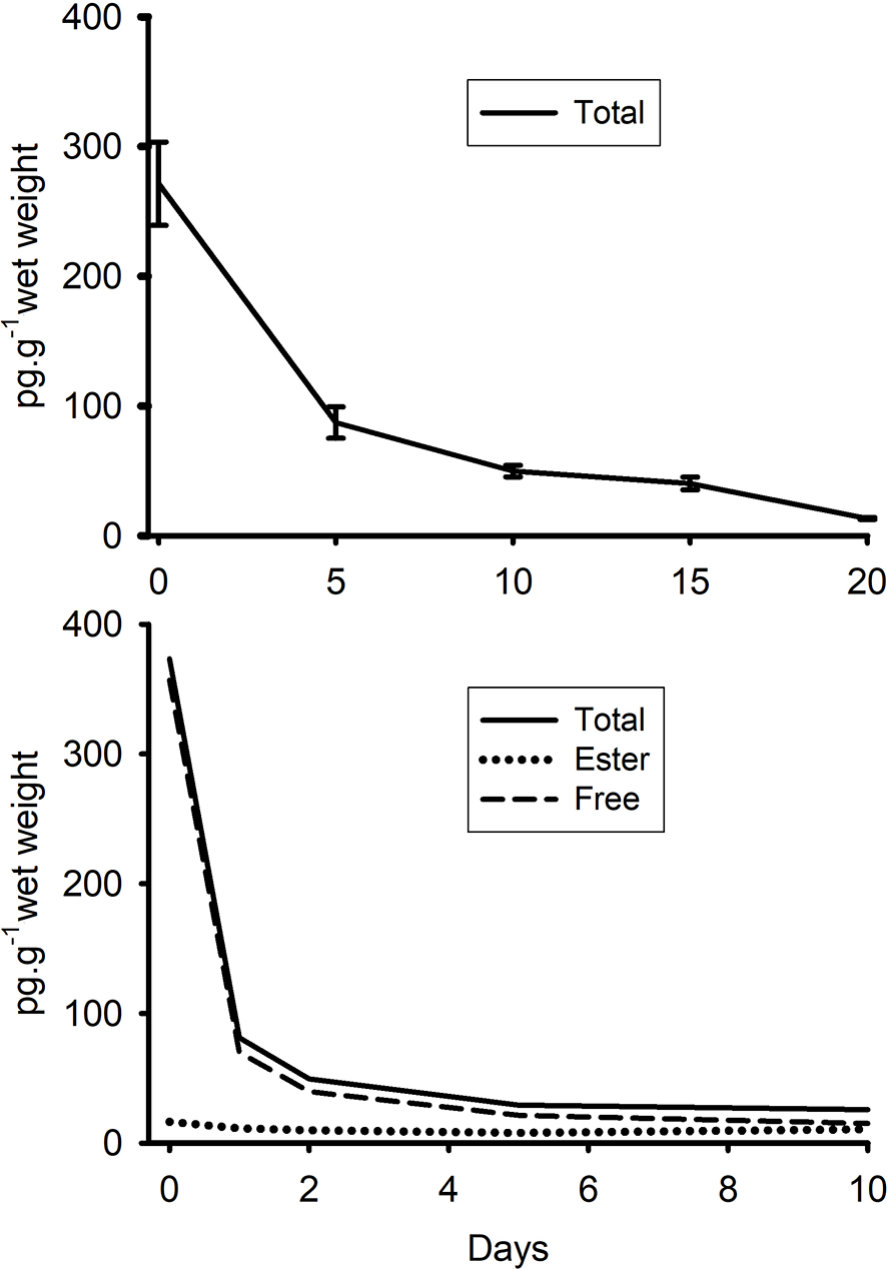
Depuration of radiolabelled EE_2_ (converted to pg g^-1^ of tissue of radiolabel) from mussels (whole tissue extracts) that had been exposed for either 96h (top graph, Exp. 1) or 24 h (bottom graph Exp. 2) to tritiated EE_2_. In Experiment 2, a portion of each extract was also partitioned twice between heptane and 80% ethanol to establish the relative proportions of esterified and free (i.e. non-conjugated) steroid. Amounts of water-soluble radiolabel were negligible. Standard errors have not been included in the second graph for clarity (as the lines were so close together).

When [^3^H]-EE_2_-treated animals were placed in clean seawater, there was a sharp drop in concentrations of radiolabel over the first five days in Experiment 1 and over 1 day in Experiment 2 (**Figure 5**), with a calculated half-life of 15 h in Experiment 2. The infrequency of sampling meant that it was not possible to get an accurate half-life for Experiment 1 (it was somewhere between 0 and 5 days!). Solvent partitioning of the extracts from Experiment 2 showed that depuration was almost entirely due to the loss of the free steroid fraction. The ester fraction, although low in amount, was relatively stable between 0 and 10 days.

### Metabolism of EE_2_

**Figure 6:**
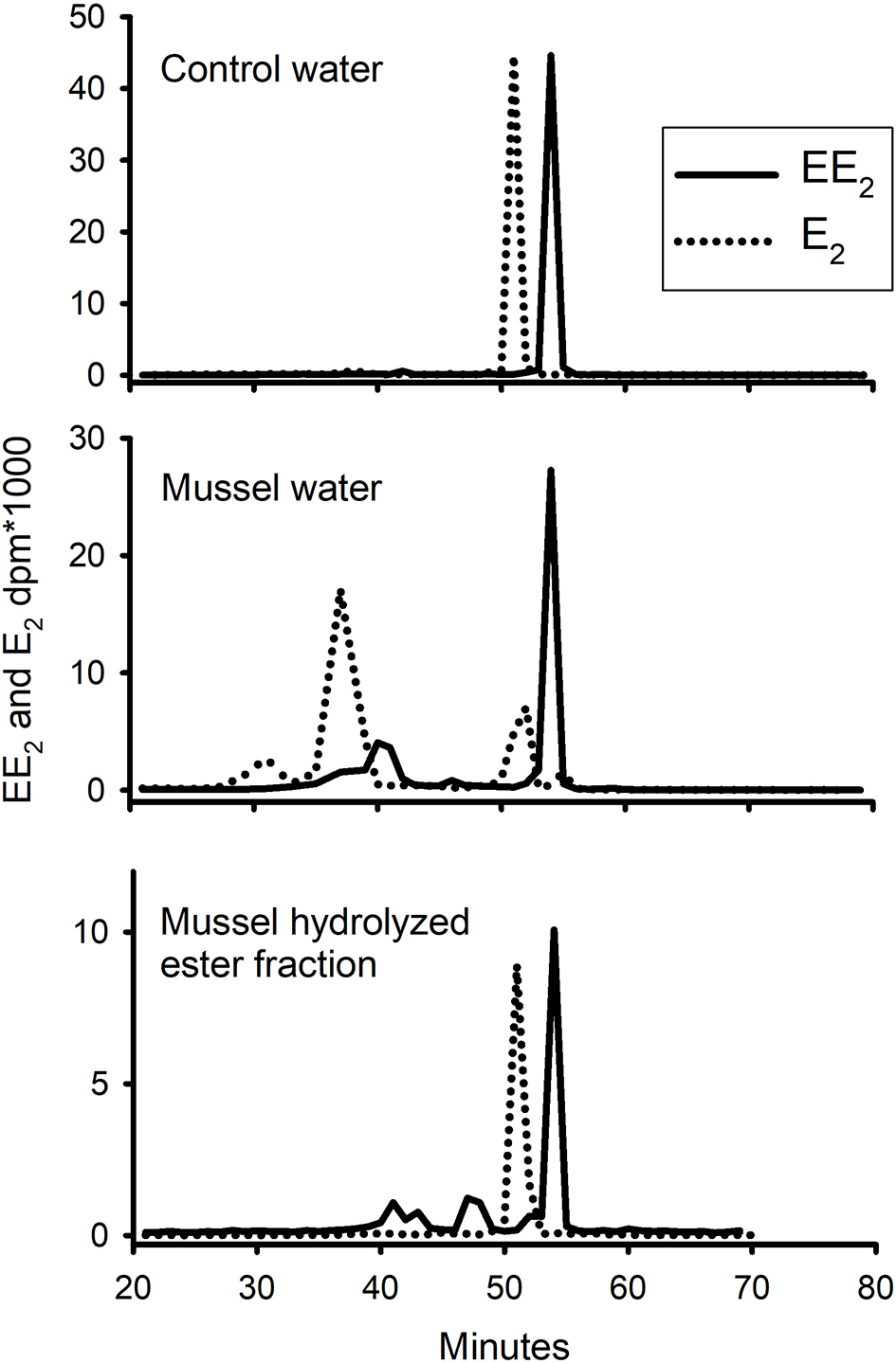
Pattern of elution of radioactive EE_2_ (solid line) and E_2_ (dotted line) on rp-HPLC extracted, after a 24h exposure period, from either sorption control (no mussels), water which contained live mussels, or hydrolysed ester fraction of the tissue. Standard EE_2_ eluted at 54 min and E_2_ at 51 min.

Water from the control tank and from the mussel tank treated with [^3^H]-EE_2_ was extracted with a C18-Seppak and separated on a rp-HPLC column (**Figure 6**). Pooled samples of the ester fraction were also saponified and the resultant hydrolysate run on the same column under the same conditions. The control water sample contained a single peak in the elution position of standard EE_2_. The water from the mussel tank contained the same peak, but also several unidentified metabolites. The hydrolysate of the ester fraction also contained some radioactivity in the elution position of EE_2_, but also had at least four other peaks (adding up to 40% of the total radioactivity). The behaviour of E_2_ in the same three treatments (taken from 8, 15) are shown for comparison. There were many peaks in the water, with the dominant peak being identified in the above-mentioned paper as 3-sulphated E_2_. There was a single peak (also previously confirmed as intact E_2_) in the ester hydrolysate.

## Discussion

We have demonstrated that mussels, when first exposed to radiolabelled steroids, take up EE_2_ as readily as E_2_. This fact should not be surprising, as EE_2_ has previously been detected in a gastropod snail (16) and specifically in mussels in at least three studies (17–19). Its uptake has also been demonstrated directly in two studies using either ^14^C-radiolabelled (20) or non-radiolabelled (21) EE_2_.

It was shown, as was found with E_2_, T and P (aforementioned papers by Schwarz and colleagues) that the amount of additional, cold EE_2_ in the water had only a weak effect on the initial rate of the [^3^H]-EE_2_ disappearance from the water and actually seemed to increase the amounts of radioactivity that could be recovered from the tissue. The important point was that the uptake of radiolabel was not ‘saturable’ by cold steroid even in amounts far higher than could be expected in the environment.

In terms of what is an ‘environmental level’ of EE_2_, Almeida et al. (19) recently tabulated a series of published studies. These showed concentrations from undetectable to 4,400 ng L^-1^. The authors, however, missed an earlier study by Hannah et al. (22), which not only tabulated a similar wide range of concentrations of EE_2_ from worldwide studies, but also analysed and discussed the fact that some of the higher concentrations that have been reported are almost certainly wrong – because if one takes into account water volumes and flow rates in the areas that were studied, the concentrations grossly exceed the amounts of EE_2_ that would have had to have been manufactured and released into those environments (i.e. the concentrations were impossible on the basis of production volumes). The authors suggested that some of these wrong values were due to methodological problems such as incomplete clean-up of samples or poor Mass Spectrometer resolution. This is probably true for some studies, however, another study (23), following an inter-laboratory exercise on the measurement of steroids in water taken from trout farms, came up with an alternative suggestion, that some of the high values were probably the result of calculation error by the scientists involved (even something as trivial as mixing up nanograms and micrograms). The authors estimated that calculation errors likely affect 30% of similar papers in the literature.

The amounts of EE_2_ radioactivity in our studies all levelled off early on and at a higher level than E_2_. In the study on E_2_ uptake by Schwarz et al. (8), the tendency to plateau was ascribed to the fact that after about 18 h, most, if not all, of free E_2_ had disappeared from the water and had been converted to stable ester (found in the tissue) and sulphate (found mostly in the water). However, in the EE_2_ uptake experiment, analysis of the water on rp-HPLC showed that at 24h that there still appeared to be a large peak of non-metabolised EE_2_. Although there were other peaks in the water, they were not substantial and there was certainly not a predominant ‘sulphate’ peak as there was with E_2_ (8). This lack of sulphate production was unexpected. However, one must bear in mind that EE_2_, like many pharmaceuticals, has been designed to resist metabolism. In fact, in the laboratory, EE_2_ has been shown to inhibit its own sulphation when present in nanomolar concentrations (24). What then brings about the apparent slowdown in uptake of EE_2_ after a few hours, even though there still appears to be a good supply of intact EE_2_ in the water? Whereas mussels avidly esterify E_2_ (8), they appear to do so at a much lower rate with EE_2_. Furthermore, even the small amount of radiolabel that does appear to be esterified turns out not to be entirely intact, as it is in the case of E_2_. The much slower ability of the mussels to esterify EE_2_ is likely because the α-ethinyl group is attached to the same carbon atom (C17) as the β-hydroxyl group that attaches to the fatty acid to form an ester (**Figure 1**).

In the absence of avid sulphation, esterification or metabolism, the most likely explanation for our results (which it must be pointed out were carried out under static conditions) is that EE_2_ uptake is plateauing because it reaches a point where its uptake is balanced by its release. At the start of exposure, the radiolabel rushes in, but then (we suggest) is held so loosely by the fats and proteins in the animal that some of it starts to come out again. In summary, mussels have high capacity for EE_2_ uptake, since uptake was not influenced much by adding up to 25 μg L^-1^ of cold EE_2_ to the water, but relatively low affinity, since the amount of radioactivity left in the water levelled off at about 85% in the 13 L tanks and 60% in the 2 L tanks. A low affinity for retaining free EE_2_ would also explain its relatively rapid depuration rate.

In regard to depuration, Ricciardi et al. (20) reported considerably longer half-lives of EE_2_ from 13 to 96 days, depending on which tissues were being investigated. What, the reader might ask, is the explanation for such a big inter-study difference? In our study, we exposed mussels for either 1 or 4 days to radiolabelled EE_2_. Although there was evidence for ester formation, the ratio of Ester : Free at the beginning of depuration was very low and what we were measuring was predominantly the depuration of free EE_2_. In their study, they exposed mussels for 38 days. After such a long time, we would predict gradual accumulation of esterified EE_2_ up to a point when there would be a noticeably higher ratio of Ester : Free at the start of the depuration period. We suggest that what the authors were measuring in their study was predominantly the eventual disappearance (subtly different from depuration) of esterified EE_2_. The authors did not distinguish between free and ester in their study. They did not mention even the possibility of esterification and just assumed that the radioactivity was all free EE_2_. This explanation is backed up by the fact that they found a half-life of only 2.7 days when they measured radioactivity in the haemolymph (plasma) of the mussels. In contrast to the tissues, one would expect the haemolymph to contain mainly free EE_2_, although possibly some ester in the form of fat droplets. The variability of the half-lives in the different tissues in their study is probably explained by the different tissues having different ratios of Free : Ester at the start of depuration. If this interpretation is correct, then this means that essentially all measurements of half-lives in both studies are wrong (in the sense that they are a mixed measure of the half-lives of two totally different compounds, one water-soluble and the other fat-soluble). This needs to be considered in any future studies on EE_2_ depuration. A more recent study (21), is also highly relevant. When mussels were treated with cold EE_2_ alone for 10 days, it was only detectable (as free EE_2_) in the tissue on day 1. However, when mixed with three other pharmaceutical compounds, it built up to 77 ng g^-1^ dry weight of tissue by day 10 and dropped to a value of 13 ng g^-1^ after a further 8 days of depuration. Perhaps this indicates one or other of these other compounds inhibit esterification – thus leading to a build-up of predominantly free EE_2_? This is only speculation.

Our uptake experiment was carried out with cortisol not because we believed it to be the stress hormone in mussels. In evolutionary terms, this would make no sense. The reason we include this steroid in our studies was because cortisol has a primary hydroxyl group at its C21 position (**Figure 1**). We hypothesised that this would be very susceptible to esterification. What we found, however, was that the mussels absorbed cortisol poorly – so we were unable to test whether or not this hydroxyl group was a suitable ligand for esterification. This finding though, recalled a study made by one of us on steroid uptake by a teleost fish (25). In that study, among a number of steroids tested, cortisol and 11-ketotestosterone (11-KT) were also notable for not being absorbed by the fish. Although this may be explained by the fact that cortisol is much more water soluble/less lipophilic (it has a substantially smaller partition coefficient (Log P), a proxy for lipophilicity, see **Table 3**), the one thing that these two steroids have in common is an oxygen group at the C11 position on the second ring of the steroid molecule (as shown in **Figure 1**). To test whether this was critical, we carried out a short experiment in which we compared the ability of the mussel to take up cortisol and 11-deoxycortisol radiolabels. In contrast to the first experiment, there was some uptake of cortisol, but the clearance rate (which may be an overestimate due to the poor fit of the data to the decay curve) was still much less than E_2_ or EE_2_, and *c*. 3 times less than 11-deoxycortisol. These results may suggest that the reduced lipophilicity imparted by an additional oxygen group at the C-11 position of cortisol has a one-way effect on the transfer across the gill membranes (whether vertebrate or invertebrate), when compared with the more lipophilic steroids studied. The reason we say ‘one-way’ is that cortisol has been shown to pass very easily from fish into the water across the gills (27). It is tempting to speculate that the hypothetical resistance to uptake *via* the same route imparted by an 11-oxygenated group might have been the driver for the evolution of cortisol as a stress steroid and 11-KT (as opposed to T) as the male androgen in teleost fish. A stress steroid and a male androgen would be of little use to fish if they could be absorbed from the water by other fishes as readily as they are excreted. This argument does not need to be applied to E_2_, because fish have evolved a high-affinity sex steroid-binding globulin in their bloodstream that strongly retains E_2_ (25). This means only small amounts of E_2_ find their way into the water in the first place.

**Table 3:** Basic information on steroids used or referred to at this study.

CAS No.	Steroid	Log P
57-63-6	17α-Ethinyl-estradiol	3.67‡
50-28-2	17β-Estradiol	4.01‡
58-22-0	Testosterone	3.32‡
57-83-0	Progesterone	3.87‡
50-23-7	Cortisol	1.61‡
52-58-9	11-Deoxycortisol	3.08‡
53187-98-7	11-Ketotestosterone	2.76†

‡VEGA-QSAR (version: 1.1.5). Experimental LogP (26).

†VEGA-QSAR (version: 1.1.5). Predicted LogP model (MLogP) 1.0.0. (26).

This study provides no new evidence on the putative biosynthesis of vertebrate steroids by mussels, so this topic will not be discussed. In regard to the possible endocrine disrupting potential of EE_2_ in mollusks, there were two highly replicated and well-funded experiments (28, 29) in which *Lymnaea stagnalis* were dosed with microgram concentrations of EE_2_ but there were no effects of any consequence. In addition, two recent studies have shown, convincingly in our view, that EE_2_ (in contrast to what it does to fishes) has absolutely no effect on egg yolk (vitellin) protein production in mussels (30, 31). With regards to other studies that have reported miscellaneous effects of EE_2_ in mollusks (32–36), we can only state that none of them was flawless - at least according to the principles of sound ecotoxicology (37). So, all in all, there is not very convincing data yet that EE_2_ in the aquatic environment has any serious effects on mussels. Whether the amounts of EE_2_ that are likely taken up by mussels, and the small amounts stored in the form of ester in the tissue, are a risk to vertebrates that eat them is hard to say. It seems unlikely, however.

## Data Availability Statement

The original contributions presented in the study are included in the article/supplementary material. Further inquiries can be directed to the corresponding author.

## Author Contributions

TS conducted the bulk of experimental work, organised data and co-authored the manuscript. AC provided critical review of the manuscript and contributed to the discussion, tables and methods sections. AS participated in the experiments, created the graph plots and a first draft version of the manuscript. IK secured funding for the work, overviewed the PhD studies (Tamar was a student at the time) and co-authored the manuscript, edited almost final versions and formatted the MS for submission. All authors contributed to the article and approved the submitted version.

## Funding

This work was funded by Defra (Grant number CB0485) and Cefas internal funds (Cefas Seedcorn).

## Conflict of Interest

The authors declare that the research was conducted in the absence of any commercial or financial relationships that could be construed as a potential conflict of interest.

## Publisher’s Note

All claims expressed in this article are solely those of the authors and do not necessarily represent those of their affiliated organizations, or those of the publisher, the editors and the reviewers. Any product that may be evaluated in this article, or claim that may be made by its manufacturer, is not guaranteed or endorsed by the publisher.
